# Patient safety culture among nurses working in Palestinian governmental hospital: a pathway to a new policy

**DOI:** 10.1186/s12913-019-4374-9

**Published:** 2019-08-06

**Authors:** Nasser Ibrahim Abu-El-Noor, Mysoon Khalil Abu-El-Noor, Yousef Zuheir Abuowda, Maha Alfaqawi, Bettina Böttcher

**Affiliations:** 1 Faculty of Nursing, Islamic University of Gaza, P. O. Box 108, Gaza, Palestine; 2 Palestinian Ministry of Health, Gaza, Palestine

**Keywords:** Patient safety, Safety culture, Adverse events, Nurses’ attitude, Medical errors Gaza strip, Palestine

## Abstract

**Background:**

Providing safe care helps to reduce mortality, morbidity, length of hospital stay and cost. Patient safety is highly linked to attitudes of health care providers, where those with more positive attitudes achieve higher degrees of patient safety. This study aimed to assess attitudes of nurses working in governmental hospitals in the Gaza-Strip toward patient safety and to examine factors impacting their attitudes.

**Methods:**

This is a cross-sectional, descriptive study with a convenient sample of 424 nurses, working in four governmental hospitals. The Attitudes to Patient Safety Questionnaire III, a validated tool consisting of 29 items that assesses patient safety attitudes across nine main domains, was used.

**Results:**

Nurses working in governmental hospitals showed overall only slightly positive attitudes toward patient safety with a total score of 3.68 on a 5-point Likert scale, although only 41.9% reported receiving patient safety training previously. The most positive attitudes to patient safety were found in the domains of ‘working hours as a cause of error’ and ‘team functioning’ with scores of 3.94 and 3.93 respectively, whereas the most negative attitudes were found in ‘importance of patient safety in the curriculum’ with a score of 2.92. Most of the study variables, such as age and years of experience, did not impact on nurses’ attitudes. On the other hand, some variables, such as the specialty and the hospital, were found to significantly influence reported patient safety attitudes with nurses working in surgical specialties, showing more positive attitudes.

**Conclusion:**

Despite the insufficient patient safety training received by the participants in this study, they showed slightly positive attitudes toward patient safety with some variations among different hospitals and departments. A special challenge will be for nursing educators to integrate patient safety in the curriculum, as a large proportion of the participants did not find inclusion of patient safety in the curriculum useful. Therefore, this part of the curriculum in nurses’ training should be targeted and developed to be related to clinical practice. Moreover, hospital management has to develop non-punitive reporting systems for adverse events and use them as an opportunity to learn from them.

## Background

In spite of advanced medical technology, continuing evidence-based research, and modern training facilities, providing safe care remains one of the major challenges in many healthcare systems [1] and patients continue to come to harm or die due to adverse events. Therefore, patient safety and safety culture became a major concern in healthcare systems and many “experts believe that healthcare quality and safety must be investigated within the framework of systems and contextual factors in which errors and adverse events occur” [2].

Patient safety was defined by the Institute of Medicine [3] as “the prevention of harm caused by errors of commission and omission,” while safety culture was defined as “the product of individual and group values, attitudes, perceptions, competencies and patterns of behavior that determine the commitment to, and the style and proficiency of, an organization’s health and safety management” [4]. In order to ensure patient safety, healthcare systems should provide an environment that is free from accidental injury through establishing operational systems and procedures that minimize the chance for errors and maximize the likelihood of intercepting them before they occur [3].

Globally, one out of 150 patients have been reported to die in hospitals as a consequence of an adverse event [5]. Varying percentages of medical mistakes have been reported in the literature. For example, a study conducted in Australia by Sorensen et al. [6] reported that 10–18% of hospitalized patients were injured by medical errors. Another study conducted by Adams and Boscarino [7], revealed that one-fifth of the people of New York in the United States were exposed to medical mistakes, while a further study from the United States showed that 35–42% of patients were exposed to medical errors [8]. Overall, one in ten patients is injured in high income countries which have sufficient funds and modern technology [9]. In contrast, it remains unclear how many patients come to harm in the healthcare systems of low income countries which have less technology, insufficiently trained staff and inappropriate infrastructure [9]. In Palestine, a survey using a global trigger tool found that one in seven patients suffer from harm and 59.3% of these had been preventable [10].

The attitude of healthcare professionals to adverse events is one of the most important components of safety culture. It is well recognized that patient safety training and education can improve patient safety attitudes and patient outcomes. Some behaviors, conditions or situations can give rise to patient harm. Addressing these factors by modifying behavior, as well as systems, can make the healthcare environment safer for patients [5]. Carruthers, Lawton [11] claimed that suitable education and training were the best strategies to improve patient safety attitudes, necessitating inclusion of patient safety in the curricula of undergraduate programs of future healthcare providers. Recently, the WHO produced a multi-professional curriculum for use in educating the healthcare professionals in this important discipline [12]. Patient safety education has been integrated in many postgraduate curricula across the world [11, 13–15]. But, so far, it only has a small presence in postgraduate education in Gaza, although several studies about safety culture had recommended incorporating it in the curricula [16–18].

In order to develop effective safety culture training programs, it is imperative that healthcare organizations assess current patient safety culture and attitudes of their employees in order to determine areas of priority [19]. In the last decades, some studies were conducted to assess attitudes of health care professionals toward safety culture in Palestine [14, 16, 20] using the Safety Attitude Questionnaire (SAQ). These studies revealed that nurses’ attitude toward safety culture was less than the cut-off point of 75. Since nurses are the backbone of the Palestinian health care system, the current study aimed to assess attitudes of nurses working in governmental hospitals in the Gaza Strip toward patient safety using the Attitudes to Patient Safety Questionnaire III (APSQ-III), which assesses attitudes on an individual basis rather than in an institutional context. Furthermore, this study examines factors that impact on nurses’ attitudes and consequently provide feasible advice to heath policy makers to improve safety culture in governmental hospitals of the Gaza Strip.

The conceptual framework that guided this study is based on Organizational Climate of Staff Working Conditions and Safety - An Integrative Model which was developed by Donabedian, Stone and colleagues [21, 22]. The model describes health care organizations in terms of structure, process, and outcomes. Organizational structure includes human resources, organizational characteristics, and physical materials. Process involves the activities and actions taken to provide health care. And outcomes are the results or changes that can be attributed to health care. According to this model, there is a dynamic interaction between the three dimensions. This interaction influences safety culture in the organization. Positive interaction between the three dimensions will result in positive outcomes/changes; while negative interactions will result in negative outcomes/changes, which will be reflected on improvement or deterioration of safety climate within that organization.

## Methods

This study aimed to assess attitudes of nurses working in governmental hospitals in the Gaza Strip toward patient safety and to examine factors that impact on nurses’ attitudes and consequently provide feasible advice to heath policymakers to improve safety culture in governmental hospitals of the Gaza Strip.

### Design, setting and sampling

A cross sectional descriptive design was used for this study. The target population was nurses who work in different departments of governmental hospital in the Gaza-Strip. A convenience sample of 424 nurses from four governmental hospitals in the Gaza Strip participated in the study. Three of these hospitals provide different services to their clients, such as medical, surgical, obstetric, and orthopedic care, while the fourth hospital provides only obstetric care. The total number of nurses working in these four hospitals is 645 nurses.

### Instrument

The instrument used in this study was a modified version of the Attitudes to Patient Safety Questionnaire III (APSQ III) [11]. This tool examines patient safety attitudes over nine factors (patient safety training received, error reporting confidence, working hours as an error cause, error inevitability, professional incompetence as an error cause, disclosure responsibility, team functioning, patient involvement to reduce error, importance of patient safety training) with ratings on a 5-point Likert scale, ranging from 1 (strongly disagree) to 5 (strongly agree). A higher score indicated a more affirmative or positive response to the factor concerned. Several items of the instrument were negatively worded and scores were reversed during analysis.

The original instrument consists of 26 items and was designed to assess the attitude of medical students toward safety culture. Four new items were added to the instrument and some modifications were done to fit the purpose of this study. The APSQ was translated into Arabic by three bilingual members of the research team with experience in health research and designing surveys. Then, face validity was assessed by faculty members of a local faculty of nursing and experienced nurses who reviewed the translation. The reviewers provided suggestions to improve the quality of the translation and to make it a better fit for its purpose. The final version of the tool was modified accordingly and then pretested on 10 nurses from the involved hospitals. These 10 nurses were excluded from the study. Scale reliability of the translated instrument was assessed using Cronbach α which revealed an acceptable value of 0.718.

### Data collection

Members of the research team, who were not working in any of the involved hospitals, handed the survey to all nurses who met the inclusion criteria at the four targeted hospitals. Inclusion criteria included nurses who hold an associate degree, Diploma, Bachelor or Masters in nursing and who have been working at their current hospital and department for the previous 6 months. After answering the questionnaire at their convenience, nurses were asked to return it to a member of the research team.

### Ethical consideration

The research protocol got the ethical approval from the human resources of hospitals at the Palestinian Ministry of Health. Each potential participant was approached by one of the research team who explained the purpose of the research for him/her. Then each participant was asked to sign an informed consent detailing the purpose of the study, the voluntary nature of participation and the confidentiality of the information gathered.

### Data analysis

Data were analyzed using the Statistical Package for Social Science (SPSS) version 18. The scores of the negatively worded survey items were reversed prior to data analysis. After data entry, the accuracy of data entry was checked by randomly double checking 10% of the returned surveys.

Before data analysis, data were examined for missing items. Questionnaires with nine or more values missing were excluded, which were seven participants in this study. In all other cases, the missing values were replaced by the mean of each variable before conducting the data analysis.

Descriptive statistics including means, standard deviations, and percentages were used for all survey items and domains. Paired sample t-tests and ANOVA were used to compare means. The Pearson correlation test was used to assess the correlation between age and years of experience and the different dimensions of the scale. These analyses used a 95% confidence interval and a significance level of 0.05.

## Results

### Characteristics of the participants

A total of 600 questionnaires were distributed to all potential participants and 431 questionnaires were returned. Seven questionnaires were excluded as they had more than nine items missing; leaving 424 nurses from four governmental hospitals with a response rate of 70.66%. The participants’ mean age was 33.05 ± 9.24 years. The majority of participants were females (56.2%), 40 years or younger (76.0%), and with a bachelor degree (62.5%). Nurses who participated in this study worked mainly in surgical departments (33.1%) followed by medical departments (29.5%). The majority of participants (57.5%) reported that they had not received previous patient safety training (Table 1).

**Table 1:** Characteristics of the respondents

Variable	F	%
Sex (*n* = 424)		
Male	190	44.8
Female	234	55.2
Age (*n* = 417) (mean = 33.05 year)		
< 30 years	177	42.4
30–39 years	140	33.6
40–49 years	59	14.1
≥ 50 years	41	9.8
Education level (*n* = 424)		
Diploma (2 years)	131	30.9
Bachelor’s degree	265	62.5
Master’s degree	28	6.6
Hospital (*n* = 424)		
Hospital 1	196	46.2
Hospital 2	93	21.9
Hospital 3	97	22.9
Hospital 4	38	9.0
Department (*n* = 420)		
Medical	124	29.5
Surgical	139	33.1
pediatrics	15	3.6
Obstetrics and gynecology	97	23.1
Others	45	10.7
Years of experience (*n* = 411)		
≤ 5 years	157	38.2
6–10	132	32.1
11–15	40	9.7
> 15 years	82	20
Received training related to safety culture (*n* = 416)		
Yes	177	42.5
No	239	57.5

*F* Frequency, % Percentage

### Attitude of nurses toward patient safety

Table 2 depicts the mean scores and standard deviations of questions assessing attitudes toward patient safety as reported by participants. The highest score was given for the item “Even the most experienced and competent doctors make errors” (4.16 points), followed by “Better multi-disciplinary teamwork will reduce medical errors” (4.09 points) and “Even the most experienced and competent nurses make errors” (4.07 points). On the other hand, the item “If people paid more attention at work, medical errors would be avoided” received the lowest score (2.12 points) followed by “Most medical errors result from careless doctors” (2.69 points) and “Learning about patient safety issues is not as important as learning other more skill based aspects of being a doctor / a nurse” (2.69).

**Table 2:** Attitude of nurses toward patient safety

Item	Mean	St.D
1. My training has prepared me to understand the causes of medical errors	3.37	1.21
2. I like my job	3.94	1.14
3. Personal input about patient care is well received at my workplace	3.58	0.96
4. Most medical errors result from careless nurses	3.58	1.23
5. Medical errors are handled appropriately my workplace	3.34	1.06
6. I would feel comfortable reporting any errors I had made no matter how serious the outcome had been for the patient	3.72	0.98
7. I don’t think I make errors	3.40	1.08
8. I feel confident I could report an error I had made without feeling I would be blamed	3.74	0.97
9. The number of hours doctors / nurses work increases the likelihood of making medical errors	3.91	1.19
10. I would feel comfortable reporting any errors other people had made, no matter how serious the outcome had been for the patient	3.56	1.01
11. I am confident I could talk openly to my supervisor about an error I had made if it had resulted in potential or actual harm to my patient	3.69	1.04
12. A true professional does not make mistakes or errors	3.66	1.12
13. Shorter shifts will reduce medical errors	3.86	1.10
14. By not taking regular breaks during shifts doctors / nurses are at an increased risk of making errors	4.05	0.98
15. Doctors / nurses have a responsibility to disclose errors to patients only if they result in patient harm	2.98	1.03
16. Patient safety issues cannot be taught and can only be learned by clinical experience when qualified	3.15	1.18
17. Even the most experienced and competent doctors make errors	4.16	0.89
18. Better multi-disciplinary teamwork will reduce medical errors	4.09	0.83
19. Patients have an important role in preventing medical errors	3.56	1.03
20. Medical errors are a sign of incompetence	3.28	1.15
21. Learning about patient safety issues is not as important as learning other more skill based aspects of being a doctor / a nurse	2.69	1.18
22. All medical errors should be reported	3.91	1.03
23. Teaching teamwork skills will reduce medical errors	4.05	0.84
24. If people paid more attention at work, medical errors would be avoided	2.12	0.94
25. Encouraging patients to be more involved in their care can help to reduce the risk of medical errors occurring	3.88	.89
26. It is not necessary to report errors which do not result in adverse outcomes for the patient	3.20	1.18
27. It is the responsibility of all health care professionals to formally report all medical errors which occur	3.71	0.97
28. Most medical errors result from careless doctors	2.69	1.08
29. Learning about patient safety issues before I qualify will enable me to become a more effective doctor / nurse	3.83	0.92
30. Even the most experienced and competent nurses make errors	4.07	0.89
Total score	3.68	0.26

### Main domains of APSQ

Table 3 depicts the mean scores and standard deviations of the main domains of the APSQ. The highest score of the nine domains was given to “Working hours as a cause of errors” (3.94 ± 0.71) followed by “team functioning” (3.93 ± 0.59). The domain that received the lowest score was “Importance of Patient Safety in the Curriculum” (2.92 ± 0.72) followed by the domain “Professional Incompetence as a Cause of Error” (3.09 ± 0.59).

**Table 3:** Descriptive statistics of main domains of APSQ

Domain	Number. of items	Mean	St.D
Patient Safety Training Received	2	3.40	1.13
Error Reporting Confidence	5	3.61	0.65
Working hours as a cause of errors	4	3.94	0.71
Error Inevitability	3	3.89	0.62
Professional Incompetence as a Cause of Error	5	3.09	0.59
Disclosure Responsibility	3	3.37	0.65
Team Functioning	3	3.93	0.59
Patient Involvement in reducing Error	2	3.73	0.70
Importance of Patient Safety in the Curriculum	3	2.92	0.72

### Association between participants’ characteristics and overall APSQ scores

Results of one-way ANOVA and t-test (Table 4) revealed that there were no statistically significant differences between the overall APSQ scores and age of participants, educational level or years of experience. The fact whether participants had received training related to safety culture or not also had no significant impact on their patient safety attitudes. On the other hand, female nurses had a slightly higher score than male nurses (*p* = 0.006).

**Table 4:** Association between participant characteristics and overall APSQ scores

Variable	Total APSQ score	SD	*P* value
Sex			0.006
Male	105.60	10.5	
Female	108.27	10.1	
Age			0.155
≤ 30 years	107.89	9.49	
> 30 years	106.48	10.87	
Education level			0.38
Diploma (2 years)	108.12	8.50	
Bachelor’s degree	106.51	10.61	
Graduate studies	107.47	10.76	
Department			> 0.001
Medical	104.95	9.30	
Surgical	105.98	10.37	
Obstetrics & gynecology	110.64	9,54	
Pediatrics	106.48	12.44	
Others	108.61	9,48	
Years of experience			0.121
≤ 5 years	107.05	10.03	
6–10 years	107.25	9.36	
11–15 years	104.92	10.16	
> 15 years	108.26	11.17	
Received training related to safety culture			0.703
Yes	107.37	9.36	
No	106.96	10.51	
Hospital (*n* = 424)			< 0.001
Hospital 1	108.15	9.34	
Hospital 2	107.49	10.77	
Hospital 3	103.21	10.45	
Hospital 4	110.31	7.74	

Furthermore, nurses displayed significant variations in their attitudes toward patient safety according to their department of work (*p* < 0.001) with nurses in obstetrics and gynecology having more positive patient safety attitudes than those in medical (*p* = 0.001) and surgical departments (*p* = 0.013). Moreover, significant differences were found in patient safety attitudes of nurses working in the four different hospitals (*p* < 0.001) with nurses working in Hospital 3 showing significantly more negative attitudes than those in the other three hospitals (Table 4). Finally, age and years of experience displayed weak correlations with different patient safety domains (Table 5).

**Table 5:** Correlation between age and years of experience and dimensions of the APSQ

		D1	D2	D3	D4	D5	D6	D7	D8	D9
Age	r	−.006	−.027	−.018	.102^a^	.068	−.032	−.133^b^	−.148^b^	.106^a^
	p	.913	.586	.717	.037	.167	.520	.006	.003	.031
Years of Experience	r	.017	−.016	.043	−.031	.083	−.017	−.062	−.110^a^	.120^a^
	p	.741	.745	.384	.534	.092	.735	.209	.026	.015

D1 = Patient Safety Training Received

D2 = Error Reporting Confidence

D3 = Working hours as a cause of errors

D4 = Error Inevitability

D5 = Professional Incompetence as a Cause of Error

D6 = Disclosure Responsibility

D7 = Team Functioning

D8 = Patient Involvement in reducing Error

D9 = Importance of Patient Safety in the Curriculum

^a^ Correlation is significant at the 0.05 level ^b^ Correlation is significant at the 0.01 level

## Discussion

In this study, the most positive attitudes to patient safety were found in the domains of ‘working hours as a cause of error’ and ‘team functioning’ with scores of 3.94 and 3.93 respectively; whereas the most negative attitudes were found in ‘importance of patient safety in the curriculum’ with a score of 2.92. Age of participants and engagement in a training course about safety culture were found not to influence nurses’ attitudes toward safety culture. On the other hand, the department and the place of work significantly influenced the reported patient safety attitudes with nurses working in surgical specialties, especially obstetrics and gynecology, showing significantly more positive attitudes towards patient safety. Similarly, two studies that were conducted in Norway [23] and Turkey [24] found a variation in nurses’ attitude toward safety culture and level of safety among different hospital departments. Another study reported that the attitude of nurses toward safety culture and their practice of safety measure was different from one hospital to another [25], suggesting varying safety cultures present in each department exerting significant influence on the patient safety attitudes of staff.

Overall, the patient safety attitudes of nurses working at governmental hospitals in the Gaza-Strip equaled 73.6% when converted to percentile. This is very close to the 75% score considered by Huang et al. [26] as a cut-off point for a positive score on the Safety Attitudes Questionnaire (SAQ). The total score reported by nurses participating in this study (73.6) is higher than the total SAQ score achieved by nurses working on the Neonatal Intensive Care Units (NICU) in the Gaza-Strip [16] and the West Bank, Palestine [14] and more than the scores reported by Elsous, Akbari Sari [20] where scores for different dimensions of SAQ ranged between 16.2 and 64.2. The differences between this study and the other studies can be related to merely using two different scales or to the fact that two of these studies [14, 16] were conducted on specialized care units, which require more attention and more sensitivity to safety culture.

So far, few studies have used the APSQ to assess nurses’ attitude as the it was originally created to assess the attitudes of medical students. However, most other studies assess patient safety attitudes in relation to specific institutions and workplaces of the participants. The advantage of the APSQ is that it is independent of the workplace and can be used for professionals working in different units or hospitals, as well as with healthcare professionals, other than medical students [11]. One of the few studies that used the APSQ to compare between nurses and postgraduate residents was conducted by Bari and colleagues [27]. Congruent with our results, their results reflected a positive attitude toward safety culture as all of the items, except one item, had a score over four (on a scale of 1–7) with the majority of items having means of over five.

Participants of this study showed the most negative attitudes to the domain entitled “Importance of Patient Safety in the Curriculum.” This might be because a large number of participants had not actually received patient safety education before or because the training they received had proven not to be reflected in clinical practice. This poses a special challenge to undergraduate and postgraduate nursing educators, which should alert nursing schools in the Gaza Strip to revise their curricula to be more inclusive of patient safety and make the delivered content more relevant to clinical practice. Moreover, the majority of participants reported that they had not received any patient safety training previously. Ironically, no statistically significant differences were found between the overall patient safety attitudes of those who had received patient safety training and those who had not. The recognition by participants in several studies of the importance of safety culture education and its inclusion in the curricula of health care professionals is prominent in the literature [13, 28–30]. But this belief has not been reflected in this study. A further explanation for this could be the experience of the ‘hidden curriculum’, which has been described by several researchers [31, 32]. It describes the fact that patient safety trainees experience a completely different clinical practice than that which they have learned in the training courses, making the course content appear irrelevant and far removed from clinical practice. This is an important issue to address for nurses educators in the Gaza Strip and requires strong collaboration between them and clinical leadership in the local hospitals.

The nurses displayed the most positive attitudes in the domains of ‘working hours as a cause of error’ and ‘team functioning.’ Other studies found that working longer hours had contributed negatively to patient safety and was associated with low point safety scores [33]. Elsous, Akbari Sari [20] found that nurses who worked 35 h per week displayed more positive attitudes to patient safety than nurses who worked more than 35 h per week. Moreover, Rogers, Hwang [34] reported a 3.3 times increased chance of making an error among nurses who worked for a longer duration (> 12.5 h shift in 24-h period). Other studies reported that tired health care professionals are at greater risk to commit errors in their practice which will endanger the lives of their clients [11, 13, 14, 16, 28, 35]. Therefore, the attitudes of the nurses in this study were in concordance with other international studies and show a realistic look at their own abilities and limitations in such conditions, which were independent of age and years of experience.

The dimension which received the second highest score (3.93) in this study was ‘team functioning’ which reflects the high impact of team functioning on safety culture. In a study conducted in Taiwan, results revealed that nurses had a positive attitude toward safety culture in their organization and that the dimension that received the highest positive response rate was “Teamwork within units”, [36]. Other studies found that nurses who had a positive attitude toward patient safety were more cooperative with their colleagues [20, 37].

Similar results were also obtained from medical students. In a study conducted by Kamran, Bari [28], medical students in Pakistan gave the highest score (6. 17 on a scale of 1–7) to ‘team functioning’. The high score given by participants in this as well as other studies, reflects the vital role of team functioning on error reduction and improving safety [13, 14, 16, 28, 38]. In the current study, the attitudes to team functioning became significantly more negative with years of experience, possibly reflecting the fact that team functioning as an important contributor to the delivery of safe care has only more recently entered the training of nurses. Therefore, nurses with less experience are more aware of this concept than the older team members. However, this supports the idea that healthcare professionals can be trained in teamwork and made more aware of its importance, which can have a direct impact on the degree of safety of delivered care [38].

However, the overall patient safety attitudes of the participants in this study showed no significant differences with increasing or decreasing age of participants. These results are congruent with the results of Abu-El-Noor, Hamdan [16], but contradicting results of others [14, 20] who found that nurses who were > 35 years old had higher scores than nurses who were under 35 years old for all dimensions of patient safety culture except for ‘stress recognition’ dimension. While Elsous, Akbari Sari [20] justified their results that older nurses are more mature and show higher responsibility in their profession which can be positively reflected on their attitude toward safety than younger nurses. This could be countered by the argument that younger nurses are more motivated and eager to learn and to be involved in training programs which may positively impact their attitude toward safety culture.

Likewise, no statistically significant differences were found between the overall patient safety attitudes in relation to years of experience of participants. This is in concordance with other studies [14, 16, 20]. However, Nabhan & Ahmed-Tawfik [39] found that safety culture was greater among nurses having less than 1 year experience in comparison with nurses who had been working for a longer time. On the contrary, our results contradict some studies that found patient safety attitude increases with years of experience [20, 40]. One study by El-Jardali and colleagues [41] found that patient safety scores were increasing with years of experience and reached their peak among those who had work experience between 11 and 15 years. Subsequently, scores started to decline with the lowest scores reported by those who had 21 or more years of experience. This could be a reflection of newly graduated health care professionals seeking advancement in their career up to a certain point, but also loss of motivation and interest thereafter, leading to a decline in their patient safety attitudes. This underlines the need for teams to consist of nurses with different levels of experience, as well as the obligation for nurse educators and trainers to provide ongoing and relevant patient safety training for nurses throughout their career.

Our results showed that female nurses had a slightly higher score in total APSQ. This contradicts the results of previous studies [14, 20, 42] which found that females had slightly lower scores than males. However, in spite of the fact that Elsous, Akbari Sari [20] reported male nurses had slightly higher scores than female nurses, the female nurses had higher score in the dimensions of ‘teamwork climate,’ ‘perception of hospital management,’ ‘working conditions and in total score.’

### Implication for practice and policy change

Nurses are the backbone of any health care system due to the nature of their work and their availability to patients around the clock. They play major roles in patient monitoring, continuity of care, providing safe environment, preventing errors and other aspects of care, as well as a vital role in maintaining and improving safety of their patients.

It is essential that nursing schools adopt a more positive attitude toward incorporating safety culture in the curricula of undergraduate and graduate nursing students. Literature revealed that the amount of education nurses obtained about patient safety improvement was positively associated with patient safety culture [24].

Hospitals also need to tailor some educational and training programs to meet their nursing staff’s needs to improve patient safety culture. Some studies showed significant improvements in most dimensions of safety culture after conducting training programs [43–45]. In a study conducted in Iran [45], results revealed that training about safety culture was found to enhance the average nurses’ safety attitudes by 44%. A significant improvement in nurses’ attitudes towards most safety culture dimensions after training was observed. The dimension which had highest improvement was “Perception of Management” (43.3%), while the dimension “Stress Recognition” showed the lowest increase (7%) following the training.

Moreover; some studies found that conducting training about safety culture and reporting errors produced a positive attitude among nurses to reporting errors and improving behavior of reporting [46]. From a different perspective, other studies found that training nurses about intra-organizational communication promoted the positive safety attitude among nurses working in hospitals [47, 48]. In a study conducted by Pettker et al. [49], nurses and other health care professionals’ who were involved in a safety culture program had experienced improvements in safety and teamwork, with significantly better congruence between doctors and nurses. Moreover, evidence-based studies found that implementing training programs about safety culture for nurses was significantly related to lower the occurrence of pressure ulcers, prolonged physical restraint, a higher frequency of event reporting which was found later on to contribute significantly to lower the occurrence of medicine errors and pressure ulcers [50].

According to Bari, Jabeen [27], lack of formal teaching may lead to inadequate reporting of adverse events and reluctance to adopting safety culture practice. Health policy makers, hospital administrators and nurse managers need to encourage nurses and other health care professionals to report adverse events in a non-punitive culture, especially as many healthcare providers avoid reporting errors to avoid facing litigation [51]. Reporting adverse events will help health organizations to analyze these events and take preventive measures so that the occurrence of similar events will be minimized in the future. In fact, in a study conducted in Iran, nurses gave the lowest score for “Non-punitive response to error” which was reflected in their evaluation to patient safety of their hospital as low [52].

According to El-Jardali, Jaafar [42], underreporting of adverse events will hinder improvement of safety culture within these organizations. In fact, a study conducted VanGeest and Cummins [53] revealed that a punitive response to error is a major barrier for disclosure of errors upon their identifications. In another study, results revealed that 47% of participating nurses had failed to report self- or coworker medication errors. The fear of punishment was found to be a major barrier for reporting medication errors. Therefore, the author suggested educating nurses about the goals of incident reporting systems to enhance patient safety culture [54]. A similar study showed that the fear of punishment, along with other reasons, was on the causes reported by nurses and doctors for not reporting adverse events and errors [55]. Therefore, in order to provide safe care and to promote positive attitude toward safety culture, health care systems and organizations need a change from blaming their personnel for their errors and use the occurrence of some errors as an opportunity to learn from it and to improve the system and prevent harm to patients.

Nurse managers and hospital administrations need to continuously communicate and give feedback about errors with their teams to improve nurses’ perception toward safety culture which in turn will help nurses to communicate more about errors and will increase the frequency of reporting adverse events [40].

Improving attitudes of health care staff toward patient safety will reduce morbidity and mortality rates and decrease length of hospital stay [26]. Therefore, improving safety culture will help to reduce expenditure of the already stretched health care system within the Gaza-Strip.

### Strengths and limitations of the study

This study has some strengths and some limitations. The strengths of this study include the large sample size and inclusion of nurses from different specialties, hospitals and geographical areas. The limitations are the fact that the study instrument was originally developed for medical students, but it was used with other health care professionals too. The use of cross-sectional design had its limitation, but the authors used it because of limited financial resources. Moreover, the use of a convenience sample limits the generalizability of the findings.

## Conclusion

Nurses working in governmental hospitals of the Gaza-Strip showed relatively positive patient safety attitudes, especially in the domains of ‘team functioning’ and ‘working hours as a cause of error’. Positive attitudes of nurses and other health care professionals are considered an indicator to providing safe care which will be reflected in reducing morbidity and mortality rates and reducing length of stay, number of hospitalization and the cost of expenditure of health services. Nurses displayed these attitudes, despite the fact that they had not received sufficient education or training related to safety culture, showing great potential for further improvement with the delivery of adequate training. Therefore; nursing schools and hospital administrators have to focus more efforts on the inclusion of patient safety culture in the ongoing nursing and other health education and training programs. Moreover, hospital management has to increase efforts on displaying leadership that demonstrates positive patient safety attitudes also in clinical practice.

## Data Availability

The datasets used and/or analyzed during the current study are available from the corresponding author upon request.

## Abbreviations

ANOVA: Analysis of variance
APSQ-III: Attitudes to Patient Safety Questionnaire III
NICU: Neonatal Intensive Care Units
SAQ: Safety Attitude Questionnaire
SPSS: Statistical Package for Social Science

## References

[1] Haerkens MH, Jenkins DH, van der Hoeven JG (2012). Crew resource management in the ICU: the need for culture change. Ann Intensive Care.

[2] Sexton JB, Helmreich RL, Neilands TB, Rowan K, Vella K, Boyden J (2006). The safety attitudes questionnaire: psychometric properties, benchmarking data, and emerging research. BMC Health Serv Res.

[3] Institute of Medicine (2009). Patient safety: achieving a new standard for care.

[4] Health, Commission S (1993). Organising for safety: Third report of the ACSNI (Advisory Committee on the Safety of Nuclear Installations) study group on human factors.

[5] de Vries EN, Ramrattan MA, Smorenburg SM, Gouma DJ, Boermeester MA (2008). The incidence and nature of in-hospital adverse events: a systematic review. BMJ Quality Safety..

[6] Sorensen R, Iedema R, Piper D, Manias E, Williams A, Tuckett A (2008). Health care professionals’ views of implementing a policy of open disclosure of errors. J Health Serv Res policy.

[7] Adams RE, Boscarino JA (2004). A community survey of medical errors in New York. Int J Qual Health Care.

[8] Blendon RJ, DesRoches CM, Brodie M, Benson JM, Rosen AB, Schneider E (2002). Views of practicing physicians and the public on medical errors. N Engl J Med.

[9] Andermann A, Ginsburg L, Norton P, Arora N, Bates D, Wu A (2011). Core competencies for patient safety research: a cornerstone for global capacity strengthening. BMJ Quality Safety.

[10] Najjar S, Hamdan M, Euwema MC, Vleugels A, Sermeus W, Massoud R (2013). The global trigger tool shows that one out of seven patients suffers harm in Palestinian hospitals: challenges for launching a strategic safety plan. Int J Qual Health Care.

[11] Carruthers S, Lawton R, Sandars J, Howe A, Perry M (2009). Attitudes to patient safety amongst medical students and tutors: developing a reliable and valid measure. Med Teacher.

[12] World Health Organization (2011). Patient safety curriculum guide: Multi-professional edition.

[13] Leung G, Ang S, Lau TC, Neo HJ, Patil NG, Ti LK (2013). Patient safety culture among medical students in Singapore and Hong Kong. Singap Med J.

[14] Hamdan M (2013). Measuring safety culture in Palestinian neonatal intensive care units using the safety attitudes questionnaire. J Crit Care.

[15] WHO, Patient Safety. Patient safety curriculum guide: Multi-professional edition. 2011.

[16] Abu-El-Noor NI, Hamdan MA, Abu-El-Noor MK, Radwan A-KS, Alshaer AA (2017). Safety culture in neonatal intensive care units in the Gaza strip, Palestine: a need for policy change. J Pediatr Nurs.

[17] Aljeesh YI, Alkariri N, Abusalem S, Myers JA, Alaloul F (2015). Staff-developed infection prevention program decreases health care–associated infection rates in pediatric critical care. J Nurs Care Qual.

[18] Aljeesh Y, Abu-El-Noor N (2015). An evidenced-based study: measuring the effect of implementing an infection control program on health care providers' compliance to infection control measures. IMPACT: J Res Appl Natural Soc Sci.

[19] Nieva V, Sorra J (2003). Safety culture assessment: a tool for improving patient safety in healthcare organizations. BMJ Quality Safety.

[20] Elsous A, Akbari Sari A, AlJeesh Y, Radwan M (2017). Nursing perceptions of patient safety climate in the Gaza strip, Palestine. Int Nurs Rev.

[21] Stone PW, Harrison MI, Feldman P, et al. Organizational Climate of Staff Working Conditions and Safety—An Integrative Model. In: Henriksen K, Battles JB, Marks ES, et al., editors. Advances in Patient Safety: From Research to Implementation (Volume 2:Concepts and Methodology). Rockville: Agency for Healthcare Research and Quality (US); 2005. P. 467-81. https://www.ncbi.nlm.nih.gov/books/NBK20497/. 21249825

[22] Henriksen K, Battles J, Marks E, Lewin D (2005). Organizational climate of staff working conditions and safety—an integrative model--advances in patient safety: from research to implementation (volume 2: concepts and methodology).

[23] Deilkås E, Hofoss D (2010). Patient safety culture lives in departments and wards: multilevel partitioning of variance in patient safety culture. BMC Health Serv Res.

[24] Turkmen E, Baykal U, Intepeler SS, Altuntas S (2013). Nurses' perceptions of and factors promoting patient safety culture in Turkey. J Nurs Care Qual.

[25] Cha BK, Choi J (2015). A comparative study on perception of patient safety culture and safety care activities: comparing university hospital nurses and small hospital nurses. J Korean Acad Nurs Adm.

[26] Huang DT, Clermont G, Kong L, Weissfeld LA, Sexton JB, Rowan KM (2010). Intensive care unit safety culture and outcomes: a US multicenter study. Int J Qual Health Care.

[27] Bari A, Jabeen U, Bano I, Rathore AW (2017). Patient safety awareness among postgraduate students and nurses in a tertiary health care facility. Pakistan J Med Sci.

[28] Kamran R, Bari A, Khan RA, Al-Eraky M (2018). Patient safety awareness among undergraduate medical students in Pakistani medical school. Pakistan J Med Sci.

[29] Shah N, Jawaid M, Shah N, Ali SM (2015). Patient safety: perceptions of medical students of dow medical college, Karachi. J Pak Med Assoc.

[30] Almaramhy H, Al-Shobaili H, El-Hadary K, Dandash K (2011). Knowledge and attitude towards patient safety among a group of undergraduate medical students in Saudi Arabia. Int J Health Sci.

[31] Liao JM, Thomas EJ, Bell SK (2014). Speaking up about the dangers of the hidden curriculum. Health Aff.

[32] Mahood SC (2011). Medical education: beware the hidden curriculum. Can Fam Physician.

[33] Wu Y, Fujita S, Seto K, Ito S, Matsumoto K, Huang C-C (2013). The impact of nurse working hours on patient safety culture: a cross-national survey including Japan, the United States and Chinese Taiwan using the hospital survey on patient safety culture. BMC Health Serv Res.

[34] Rogers AE, Hwang W-T, Scott LD, Aiken LH, Dinges DF (2004). The working hours of hospital staff nurses and patient safety. Health Aff.

[35] Leung GKK, Patil NG. Patient safety in the undergraduate curriculum: medical students' perception. Hong Kong Med J. 2010;16(2):101–5. 20354243

[36] Chen I-C, Li H-H (2010). Measuring patient safety culture in Taiwan using the hospital survey on patient safety culture (HSOPSC). BMC Health Serv Res.

[37] Lee NJ, Kim J-H (2011). Perception of patient safety culture and safety care activity among nurses in small-medium sized general hospitals. J Korean Acad Nurs Adm.

[38] Wetzel AP, Dow AW, Mazmanian PE (2012). Patient safety attitudes and behaviors of graduating medical students. Eval Health Professions.

[39] Nabhan A, Ahmed-Tawfik M (2007). Understanding and attitudes towards patient safety concepts in obstetrics. Int J Gynecol Obstet.

[40] Ammouri A, Tailakh A, Muliira J, Geethakrishnan R, Al Kindi S (2015). Patient safety culture among nurses. Int Nurs Rev.

[41] El-Jardali F, Dimassi H, Jamal D, Jaafar M, Hemadeh N (2011). Predictors and outcomes of patient safety culture in hospitals. BMC Health Serv Res.

[42] El-Jardali F, Jaafar M, Dimassi H, Jamal D, Hamdan R (2010). The current state of patient safety culture in Lebanese hospitals: a study at baseline. Int J Qual Health Care.

[43] Ginsburg L, Norton PG, Casebeer A, Lewis S (2005). An educational intervention to enhance nurse leaders' perceptions of patient safety culture. Health Serv Res.

[44] AbuAlRub Raeda Fawzi, Abu Alhijaa Eyad Hani (2014). The Impact of Educational Interventions on Enhancing Perceptions of Patient Safety Culture Among Jordanian Senior Nurses. Nursing Forum.

[45] Azimi L, Tabibi SJ, Maleki MR, Nasiripour AA, Mahmoodi M (2012). Influence of training on patient safety culture: a nurse attitude improvement perspective. Int J Hosp Res.

[46] Kim M (2010). The effectiveness of error reporting promoting strategy on nurse's attitude, patient safety culture, intention to report and reporting rate. J Korean Acad Nurs.

[47] Kim KJ, Han JS, Seo MS, Jang BH, Park MM, Ham HM (2012). Relationship between intra-organizational communication satisfaction and safety attitude of nurses. J Korean Acad Nurs Adm.

[48] Jeong PS, Yeon KJ, Ock LY (2012). A study on hospital Nurses' perception of patient safety culture and safety care activity. J Korean Critical Care Nurs.

[49] Pettker CM, Thung SF, Raab CA, Donohue KP, Copel JA, Lockwood CJ (2011). A comprehensive obstetrics patient safety program improves safety climate and culture. Am J Obstet Gynecol.

[50] Wang X, Liu K, L-m Y, J-g X, H-g H, L-f Z (2014). The relationship between patient safety culture and adverse events: a questionnaire survey. Int J Nurs Stud.

[51] Alahmadi H (2010). Assessment of patient safety culture in Saudi Arabian hospitals. Qual Saf Health Care.

[52] Bahrami MA, Chalak M, Montazeralfaraj R, Tafti AD. Iranian nurses’ perception of patient safety culture. Iran Red Crescent Med J. 2014;16(4):e11894. https://www.ncbi.nlm.nih.gov/pmc/articles/PMC4028756/. 10.5812/ircmj.11894 PMC4028756 24910783

[53] VanGeest JB, Cummins DS. An educational needs assessment for improving patient safety. White Paper Report. 2003;3. http://www.academia.edu/download/42302097/An_Educational_Needs_Assessment_for_Impr20160207-28266-bf13is.pdf.

[54] Chiang H-Y, Lin S-Y, Hsu S-C, Ma S-C (2010). Factors determining hospital nurses' failures in reporting medication errors in Taiwan. Nurs Outlook.

[55] Madsen M, Østergaard D, Andersen H, Hermann N, Schiøler T, Freil M (2006). The attitude of doctors and nurses towards reporting and handling errors and adverse events. Ugeskr Laeger.

